# Meanings and vulnerability to HIV/AIDS among long-distance truck drivers in Brazil

**DOI:** 10.1590/S1518-8787.2016050006185

**Published:** 2016-11-24

**Authors:** Laio Magno, Marcelo Eduardo Pfeiffer Castellanos

**Affiliations:** I Departamento de Ciências da Vida. Universidade do Estado da Bahia. Salvador, BA, Brasil; II Instituto de Saúde Coletiva. Universidade Federal da Bahia. Salvador, BA, Brasil

**Keywords:** Transportation, manpower, Acquired Immunodeficiency Syndrome, ethnology, Anthropology, Health Vulnerability, Gender and Health, Interpersonal Relations, Man’s Health

## Abstract

**OBJECTIVE:**

To understand the meanings assigned by long-distance truck drivers to HIV/AIDS and its transmission and prevention, bearing in mind different contexts of vulnerability.

**METHODS:**

Qualitative research with 22 truck drivers. Semi-structured interviews and participant observation were conducted in highways of the state of Bahia in 2013. We selected male truck drivers, with one year or more of work experience in long-distance routes. We carried out the thematic analysis of the interviews, to identify different contexts of vulnerability.

**RESULTS:**

The results showed that the insertion of truck drivers in contexts of high social vulnerability (poor working conditions, violence on the roads, and use of alcohol and other drugs) along with the advances in access and effectiveness of treatment for AIDS promote a reduced perception of the risk and severity of this disease. In addition, the notion of “risk group” and the symbolic division between “home space” (protected) and “street space” (unprotected) intensified a restricted and specific use of condoms, guided by the opposition between “woman of the street” (unknown women, prostitutes, among others) and “woman of the house” (wives, girlfriends).

**CONCLUSIONS:**

The meanings assigned by truckers to AIDS incorporated elements of recent transformations of the expanded social context, such as the development of health technologies (especially anti-retroviral drugs) and the guarantee of free access to treatment in the Brazilian public health system; but also incorporated old elements of social vulnerability context – such as the poor working conditions on Brazilian highways.

## RESUMO

**OBJETIVO:**

Compreender os significados atribuídos pelos caminhoneiros de rota longa ao HIV/aids e à sua transmissão e prevenção, tendo em vista diferentes contextos de vulnerabilidade.

**MÉTODOS:**

Pesquisa qualitativa com 22 caminhoneiros. Foram realizadas entrevistas semi-estruturadas e observação participante em rodovias do estado da Bahia em 2013. Foram selecionados caminhoneiros do sexo masculino, com um ano ou mais de experiência de trabalho em rotas de longa distância. Realizou-se análise temática das entrevistas, orientada para identificação de diferentes contextos de vulnerabilidade.

**RESULTADOS:**

Os resultados mostraram que a inserção dos caminhoneiros em contextos de alta vulnerabilidade social (más condições de trabalho, violência nas estradas e uso de álcool e outras drogas) e os avanços no acesso e efetividade do tratamento para aids favorecem a minimização da percepção de risco e gravidade dessa doença. Além disso, a noção de “grupo de risco” e a divisão simbólica entre “espaço da casa” (protegido) e “espaço da rua” (desprotegido) intensificaram um uso restrito e específico do preservativo, orientado pela oposição entre “mulher do mundo” (desconhecidas, prostitutas, entre outros) e “mulher de casa” (esposas, namoradas).

**CONCLUSÕES:**

Os significados atribuídos pelos caminhoneiros à aids incorporaram elementos de transformações recentes do contexto social ampliado, como o desenvolvimento de tecnologias em saúde (com destaque para os antirretrovirais) e a garantia de acesso gratuito ao tratamento no sistema público de saúde no Brasil; mas também incorporaram antigos elementos do contexto de vulnerabilidade social – a exemplo das más condições de trabalho nas estradas brasileiras.

## INTRODUCTION

The AIDS pandemic still represents a major challenge regarding the fight against the stigma and criminalization of sexual minorities, the access to HIV prevention and treatment, and the greater attention to socially vulnerable groups^16^ ^,^ ^a^.

The Joint United Nations Programme on HIV/AIDS indicates that people who live in extreme mobility – military, truck drivers, among others – may experience high vulnerability contexts to infection by HIV^a^.

The largest permanence on road seems to increase the chance of HIV infection in long-distance truck drivers^5^. Studies on Brazilian truck drivers show that, in their travels, they relate to casual partners^6^ ^,^ ^13^ ^,^ ^24^ or sex professionals^6^. The option for unprotected sex^6^, based on the physical appearance of the partner^11^, and the “macho culture”^24^ aggravate this situation.

Structural work-related factors also seem to favor the drivers’ vulnerability to HIV, even if indirectly. According to Sastry^19^, although many studies show the importance of structural factors to the risk of HIV infection, yet there is little empirical researches dealing with this issue in truck drivers. In analyzing narratives of truck drivers in Indian highways, the author found a context of socially vulnerability, marked by marginalization, work informality, violence, and poor conditions of work, with serious consequences for the prevention of HIV/AIDS among truck drivers.

In Brazil, studies show the existence of poor working conditions^1^ ^0,^ ^23^, high workload imposed by companies and shipping companies^18^, use of amphetamines^10^ ^,^ ^13^ ^,^ ^14^, and consumption of alcohol and other drugs^21^ among truck drivers.

These situations are aggravated on a general framework of limitation of health policies and programs that deal with such situations, directly or indirectly, reinforcing the idea that truck drivers experience different contexts of vulnerability. This article aims to understand the meanings assigned by long-distance truck drivers to HIV/AIDS and its transmission and prevention, bearing in mind different contexts of vulnerability.

## METHODS

We conducted a qualitative research with 22 truck drivers. Semi-structured interviews and complementary field observations were both performed between April and August of 2013. The 22 interviews were conducted outdoors (guided by a interview script), audio recorded, and transcribed. The saturation criterion was adopted to define the number of interviews. We selected male truck drivers, with one year or more of work experience in long-distance routes (i.e., involving three or more states in Brazil).

The studied population recruitment, interviews, and observations were carried out at the following locations: 1) on the port area of Salvador; 2) in a gas station and a private parking lot on Highway Br 324 (region of Simões Filho, Bahia); 3) in a courtyard of a cargo company in Feira de Santana, BA. The three locations have a high concentration of long-distance route truck drivers.

The design of the research problem, field work, and data analysis were based on assumptions of interpretive anthropology^8^ theory and of vulnerability concept ^2^ ^,^ ^12^.

Ayres et al.^2^ argue that the concept of vulnerability is emerging in the field of public health and is characterized by “a set of individual and collective aspects related to the greater susceptibility of individuals and communities to a disease or injury and, inseparably, lower availability of resources for their protection” (p. 78).

The focus of vulnerability in health seeks to explore different levels of analysis of the social determination of the health-disease-care process, with particular attention to the relationships between private situations and specific social contexts. A widely used formulation^12^ in relation to AIDS provides three levels of analysis: individual vulnerability, outlined by physical, cognitive, and behavioral factors related to a health problem that affects the individual; programmatic vulnerability, outlined by the performance of policies, programs, and services as intermediary elements between particular situations experienced by individuals and wider social contexts that favor or not the access to social rights and protection actions; social vulnerability, expressed by the operation of culture, religion, morals, politics, economics, among others, in determining the health-disease-care process. It assumes that contexts characterized by violating or weakening the human and social rights tend to increase vulnerability contexts in the three levels of analysis.

Geertz^8^ defines culture as webs of meaning triggered and reworked in social interactions. We assume that such “webs” are present in the contexts of vulnerability to HIV/AIDS, calling upon analysis that overcome the mere identification of individual risk behaviors, for example.

The data generation and its analysis were performed keeping in mind that the meanings assigned by truck drivers to HIV/AIDS (and to its transmission and prevention) were strongly related to social performances – related to gender, sex, and work. These performances were considered in specific social interaction contexts, incorporating elements of the broader social contexts in which they were inserted. The analysis of these meanings and interpretations aimed to identify contexts of vulnerability to HIV/AIDS.

The thematic analysis identified three main themes: “today there are much worse diseases”, “taking home a disease (...) I’m really afraid”, and “risk groups, woman of the house, and condom”. This research was approved by the Research Ethics Committee of the Institute of Collective Health (Process 280,068/2013).

## RESULTS

Most of the 22 respondents had 36 years of age or older, stable partner, low educational level, and more than 10 years working as a truck driver. Regarding the place of residence, there was a high concentration in the Southeast and Northeast regions.

The three research sites were characterized by intense male sociability, structured by activities, values, and work relations belonging to the universe of truck drivers. Gas station attendants, “*chapas*”^b^, and female workers at restaurants are characters acting in these social interactions. Although we did not carry out observations at night, respondents reported the presence of prostitutes and shemales in such places at night.

The observations and interviews showed that truck drivers consider gas stations as places to the care of the body; whilst the waiting places for loading and unloading (port and company) did not present a suitable structure for accommodation of drivers. These places were associated with difficult negotiations about the freight and with the increased time that drivers remain away from their homes.

The interviews showed that the contexts of vulnerability of truck drivers to HIV/AIDS are outlined in different levels, characterized by the following elements: the notion of risk group, the use of the opposite categories “house/street” and the performances of gender characterized by the hegemonic masculinity, altogether composing the individual vulnerability; the reduced concerns with AIDS (“optimism”) related to the access to effective treatments, composing a “reverse effect” of programmatic vulnerability; the poor working conditions, common criminal violence present on long routes, and the disregard of the public power, altogether overlapping the health concerns and reinforcing a negative view about the State, composing the social vulnerability. These contexts of increasing vulnerability to HIV/AIDS are strongly experienced by long-distance truck drivers.

### Minimizing HIV/AIDS: “today, there are much worse diseases”

Some respondents consider AIDS as a “half concealed taboo” that can lead to “embarrassment”. Sometimes, they even characterize it as “violent”, “killer”, “bad”, “dangerous”, or even comparable to “cancer”.

However, for most, the concern over HIV/AIDS was extremely minimized before other adversities faced on the roads.

[The biggest concern of the truck driver] today, is the problem of robbery. (r. 8, 72 years old)

There’s no condition, none at all. (...) you have to drink water every four hours. But there’s no water fountain! Where am I going to stop the truck? If we have to park the truck (...) there’s no bathroom, there’s nothing, man! (r. 18, 32 years old)

Our government, rulers, they don’t look, they don’t value the truck driver. Driver to them is not a profession. Got it?! It’s not a profession. (...) The police wants to extort us. (r. 19, 44 years old)

The low concern with HIV/AIDS was enhanced by the minimization of its gravity, by a new range of meanings related to the assumptions of its low lethality and the good quality of life of people living with the disease and under treatment.

There are few people I know that today live very well with AIDS, but in the past it was (...) the bogeyman of all. (...) They are people from my city. (r. 10, 51 years old)

These new ideas about AIDS are articulated with a sort of old social representations. If, before, this disease was seen widely as a “bogeyman”, now it begins to be regarded as “lighter” than other “worse” diseases – such as cancer, mentioned by some respondents.

### “Taking home a disease (...) I’m really afraid”

The notions of danger and risk were identified in the statements about sexual, family, and work performances as elements of gender identity. Thus, for some, this situation was expressed in the open confrontation of road risks and of unprotected sex. Most of them addressed the fear of “taking” the disease to the family, strengthening the meaning of AIDS as a moral threat. The figures of “adventurer man” and “father of the family” were significant references in these social performances.

Two respondents described personal situations of unprotected sex, justifying such an option by the emotional stimulus of exposing themselves to the risk:

The adrenaline is much stronger (...) Think about the feeling of putting your dick at stake (laughs) (...) I won’t tell you that I’m not gonna do it without a condom. For sure, tomorrow or after, I will be doing it again without. (laughs). (r. 18, 32 years old)

We observe a mocking tone of the interviewee with the interviewer, creating a mood of complicity (“conversation between men”) when he speaks of his sexual adventures. This is a narrative performance, taken by one of the younger respondents, who seeks to highlight the figure of the irresponsible “adventurer” as an important element of his manhood.

Gender identity admits different performances and feelings. Therefore, the same respondent continues:

Many and many times, I don’t remember (of condoms) or think that the woman is very hot and end up not using (...) But when we fuck someone without a condom (...) we worry about two weeks (...) (in) taking home a disease, I’m really afraid of it. But I’m gonna do it, what’s done is done! (r. 18, 32 years old)

Married and with a son, this respondent oscillates between the self-image of “adventurer” and “father of the family” – therefore, the one that can threaten the “home”, but who must protect it.

For many respondents, the “self care” was meant as “care with the house/family”, as a strategy for protection against “external threats”: “You get one of those hookers, get home, deposit on your wife, you sick with AIDS! (...) It’s not good” (r. 14, 65 years old).

We see that this male morality triggers a clear division between “home” and “street”. In each of these spaces, the respondents admit different behaviors that act as symbolic borders. These borders must not be blurred, thereby avoiding the introduction of a (reprehensible) element of the street (disease, condom) within the home space.

### Prevention strategies: “risk groups, woman of the house, and condom”

We have seen that minimizing the severity of AIDS did not prevent its meaning as a threat. However, this threat is bounded by the notion of “risk group” and by the category of “street” (as opposed to “home”). So, it is in the middle of this range of meaning that the protection strategies, stated by the respondents, gain sense and orientation – leading towards the adoption of exclusive (but unprotected) sex with the “woman of the house”, or even the use of condoms with “women of the street”.

People identified by respondents as belonging to “risk groups” still refer to the classic groups identified by epidemiologists during the initial phase of the epidemic, in the late 1980s: homosexuals, drug users, and sex professionals. Respondents exclude “women of the house” and themselves from these groups, indicating low self-perception of risk.

She (wife) has the confidence that I am on the road, but alone. And I also have confidence that she also respects me and is alone. (...) We are not (...) part of the risk group (...) they are people who use drugs (...) with many partners. (r. 10, 51 years old)

Therefore, avoiding sexual relations with several people – especially, “prostitutes, women of the street, and fags” –, restricting them (almost) exclusively to the “woman of the house”, was considered by respondents as a good preventive strategy, even if it is of difficult execution.

I think it prevents [AIDS], when you don’t go out with any woman but the woman of the house. (...) Through the woman one gets it too, but from the fag is more guaranteed. (r. 7, 49 years old)

Respondents categorize subjects and situations, present in their contexts of social interaction, to measure different degrees of risk. The category “woman of the house” is not limited only to the wife, also involving women that have specific attributes of the space of the “house”. This is, therefore, a “language of relations” (more than substantive attribute!) – as Goffman advocates about stigma –, a language produced in a broader web of meanings.

One of the respondents, for example, does not use condoms in the extramarital relationship with a “girlfriend”. The fact that the “girlfriend” is married to another man (taking the place of “woman of the house”), in addition to the long term “dating”, justifies for him the unprotected sex.

When I’m dating sometimes it passes, without a condom. But not anyone (...) There’s a woman ... but I know her for nine years (...) Sometimes, I don’t use condom, no. But if I get a woman I don’t know, I have to use. (...) She’s a married woman. (...) I always pass by there, I see her all the time. (r. 12, 54 years old)

We see that the use of condoms, although not consistent, is more related to the space of the “street”, as a way to meet the so-called “men’s needs” for sex, possibly more present in long routes. Many of the women who populate the contexts of social interaction of truck drivers, especially those involved with sexual service, are considered to be “anyone”; that is, as someone without bonds and who has no major concerns with the risk of infection by diseases – “rotten women” in the words of a interviewee, clearly denoting the stigma present there.

I only don’t use (condom) with a wife that I had (...) and with my current one (...) But (...) a random woman, or even a girlfriend out of the house, I never stopped using. It is for fear of getting some disease, a pregnancy and an allowance to pay for. (...) My wife knows me, I know her, we don’t use. There is (...) no danger. (r. 1, 63 years old)

The use of condoms was also related to the prevention of unwanted pregnancies, inside and outside home.

## DISCUSSION

The results show that the respondents did not ignore the existence of AIDS. However, elements of the social context of truck drivers are a priority in their field of concerns, decreasing the perception of risk to HIV/AIDS. This situation might affect negatively the adoption of individual protection strategies and strengthen the low search for health services by the male population^7^.

Insecurity and risk are elements consistently present in the working context of truck drivers, related to the roads without care and without appropriate support structure, to the pressure of companies, to contexts of violence, and drug addiction and prostitution intensely present on long routes. Truck drivers feel a great neglect of the State, expressed by its omission before such insecurity and by the merely supervising action of the police. This is, therefore, a strong context of increasing vulnerability to HIV/AIDS, because it decreases the health attention and expectations in relation to social rights (decent work, health etc.) and produces specific exposures to the virus.

The high workload and lack of proper structure in the stopping places is widely reported by studies in Brazil^15^ ^,^ ^18^ ^,^ ^21^ and in India^19^. The pressure of shipping companies for delivery of loads, many hours of work per day, and the lack of defined work shifts is usually associated with the use of amphetamines^14^ ^,^ ^15^ ^,^ ^18^. The use of these substances, in turn, is related to the higher chances of unprotected sex among truck drivers^6^.

The meanings assigned by truck drivers to HIV/AIDS have incorporated changes that took place in the development of health technologies (especially anti-retroviral drugs), in the world context, and transformations in health policies in Brazil, with emphasis on the free universal access to AIDS treatment in the Brazilian Unified Health System. We identified the reduction of the perception of severity of AIDS, by this incorporation, as a “reverse effect” of the access to treatment. This effect can act in increasing the programmatic vulnerability to the prevention of HIV infection. The existence of known people living (“well”) with HIV/AIDS reinforces this reduced perception.

Visions that minimize the severity of AIDS have been identified in other studies^22^, configuring a phenomenon called “optimism”. This “optimism” may lead to the loosening of personal surveillance on sexual behaviors considered “of risk”.

We believe that such “loosening” should not be seen as an individual isolated “behavior” or sensitive to only one contextual element (more extensive offer of effective treatment, for example), But it should be understood in the complex social dynamics related to the production of different contexts of vulnerability to HIV/AIDS. In this sense, the lack of policies and programs specifically aimed at truck drivers on the national scene, on one hand, and – perhaps even more problematic – the historic inattention of the government to male specificities and their implications for men’s health, on the other, certainly participate in the abovementioned “loosening” process. A health policy aimed at men came to light only recently, and programs specifically directed to mobile populations still do not exist, in Brazil.

The meanings assigned by truck drivers to AIDS (and the perception of risk/protection) are mediated both by broader social contexts and by social interaction contexts, along and beyond the roads.

In social interaction contexts, relevant symbolic spaces to the meaning of HIV/AIDS are demarcated. The delimitation of spaces of familiarization of sexual and affective relations, in contrast to spaces marked by mistrust, violence, and drugs, appeared as an organizing axis of the social experiences of respondents, affecting their perceptions of risk and protection strategies for HIV/AIDS. In this sense, these experiences can be considered as important elements in the context of individual vulnerability of those subjects to HIV/AIDS, not being reduced to an individual behavior isolated from broader social contexts.

The limited and specific use of condoms declared by the respondents involves both the perception of safety in relation to the “woman of the house”, resulting from a relation of trust and symbolic proximity, and the perception of risk, marked by the mistrust and symbolic distance maintained in relation to the “woman of the street”. These perceptions are measured in gender performances that seek to assign different meanings to manhood (“caregiver”, “adventurer”, “responsible”, among others). Studies indicate low condom use with fixed partners among truck drivers^24^ and the greater acceptance of its use among men in general^7^ ^,^ ^20^ in casual sexual relations with women regarded as “unknown”, as opposed to fixed or “known” partners.

This is a complex situation that requires reflection on social and cultural aspects of the functioning of Brazilian society, as indicated by Da Matta^3^ ^,^ ^4^ and Parker^17^. “House” and “street” are regarded as “social significance realms” with particular world views or ethics, not only as geographic locations. The place given to the “girlfriend” on long route exemplifies this idea and shows how truck drivers update in their gender performances the distinction between house and street.

The “world of the house” (private) is commonly related to the space of family, affection, care, personal relationships, tradition, and values, while the “world of the street” (public) is directed to the place of movement, work, insecurity, individuality, impersonality, and leisure. Truck drivers are on the move, for long periods and routes. In some situations, openly facing the risk is an important element of their gender performance. In others, this performance is attached to the construction of spaces of “familiarity”, as in the relations with the “girlfriends”, for example.

The narratives of Indian truck drivers about sexuality had a similar division between the “house”, representation of monogamous and safe sex, and the “street”, which referred to the commercial sex with sex professionals^19^.

In the statements of our study, we also observed the persistence of old ideas about prevention and transmission, as the classic “risk groups” and “deviant behaviors”, identified also by Herzlich and Pierret^9^, when investigating news on AIDS published in French newspapers of the early 1980s. These are new meanings and old ideas that work on minimizing risk perception, whenever the individuals identify themselves and their practices as distanced from those “groups” and “behaviors”.

By the data here produced, we were able to identify different contexts of vulnerability to HIV/AIDS among truck drivers, but, in spite of this, the information provided by other data sources and types of study may (re)situate the results and discussions presented here.

More general elements related to the social context, work, public actions, technologies, and health policies interrelate with cultural elements that guide the performances and gender relations in specific situations experienced along the truck drivers’ route. The long route intensifies the insecurity and risk, at the same time in which requires familiarization strategies of some spaces and characters that make up the interaction contexts of the investigated truck drivers. Our study showed how relations are established between meanings, performances, and social contexts responsible for the increasing vulnerability of truck drivers to the transmission of HIV/AIDS. Elements of gender/masculinity, occupation/working conditions, and violence are highlighted in these relations, articulating in different contextual levels of vulnerability.

Thus, this study contributes to the discussion about vulnerability to HIV beyond purely behaviorist analyses, mainly when it shows the complexity of issues related to a key population group to AIDS prevention policies.

The Brazilian Ministry of Health has conducted important partnerships with SEST/SENAT (Social Service of Transport and National Transport Learning Service) with the goal of developing health prevention interventions, such as condom distribution, provision of vaccines and rapid tests for HIV and viral hepatitis. However, often, these actions are punctual and not widely articulated with the health-care network; also, they do not consider the broader social context experienced by this population.

Thus, our study reinforces the need for programmatic actions of HIV/AIDS prevention and health promotion, systematic and well articulated to the health care systems, considering the specificity of the mobility experienced by long-distance truck drivers. These actions must involve the organization of health services in an appropriate way to meet the needs of people in a situation of high geographical mobility. We also defend intersectoral programs and policies to promote health and safety, acting effectively on the contexts of social vulnerability identified in this study. Such actions must be guided by ensuring the right to health and decent work, by public policies attentive to contexts of social vulnerability.

## Footnotes

a UNAIDS - Joint United Nations Programme on HIV/AIDS. The gap report. Geneva; 2014 [cited 2014 Dec 2]. Available from: http://www.unaids.org/sites/default/files/media_asset/UNAIDS_Gap_report_en.pdf

b
*Chapas* are men at the roadside who guide truck drivers and provide general services. Several truck drivers from other regions are guided and assisted by them when they arrive in a new city.
